# Construction of K^+^ responsive surface on SEBS to reduce the hemolysis of preserved erythrocytes†

**DOI:** 10.1039/c8ra08215d

**Published:** 2019-02-11

**Authors:** Xingkun Luan, Haozheng Wang, Zehong Xiang, Jiruo Zhao, Ying Feng, Qiang Shi, Baijun liu, Yumei Gong, Shing-Chung Wong, Jinghua Yin

**Affiliations:** Shandong Provincial Key Laboratory of Olefin Catalysis and Polymerization, Key Laboratory of Rubber-Plastics (QUST), Ministry of Education/Shandong, Qindao University of Science and Technology Qingdao 266042 P. R. China yingfeng@qust.edu.cn; State Key Laboratory of Polymer Physics and Chemistry, Changchun Institute of Applied Chemistry, Chinese Academy of Sciences Changchun 130022 P. R. China shiqiang@ciac.ac.cn; Alan G. MacDiarmid Institute, Jilin University Changchun 130026 China; School of Textile and Material Engineering, Dalian Polytechnic University Dalian 116034 P. R. China; Department of Mechanical Engineering, University of Akron Akron Ohio 44325-3903 USA

## Abstract

Hemolysis of stored erythrocytes is a big obstacle for the development of new plasticizer-free polymer containers. Hemolysis is mainly caused by cell membrane oxidation and cation leaks from the intracellular fluid during storage. To construct an anti-hemolytic surface for a plasticizer-free polymer, we fabricated 2-*O*-α-d-glucopyranosyl-l-ascorbic acid (AA-2G)-loaded polycaprolactone (PCL)-crown ether micro/nanofibers on the surface of styrene-*b*-(ethylene-*co*-butylene)-*b*-styrene (SEBS). Our strategy is based on the sensitive response of the crown ether to leaked potassium, causing the release of AA-2G, the AA-2G can then remove the excess ROS, maintaining the Na/K-pump activity and the cell integrity. We demonstrated that the PCL-crown ether micro/nanofibers have been well prepared on the surface of SEBS; the micro/nanofibers provide a sensitive response to excess K^+^ and trigger the rapid release of AA-2G. AA-2G then acts as an antioxidant to reduce the excess ROS and maintain the Na/K-pump activity to mitigate cation leaks, resulting in the reduced hemolysis of the preserved erythrocytes. Our work thus provides a novel method for the development of plasticizer-free polymers for the storage of erythrocytes, and has the potential to be used to fabricate long-term anti-hemolytic biomaterials for *in vivo* use.

## Abstract 2

A K^+^ responsive surface was constructed on a SEBS substrate to release anti-oxidants to reduce hemolysis of the preserved red blood cells.

## Graphical abstract

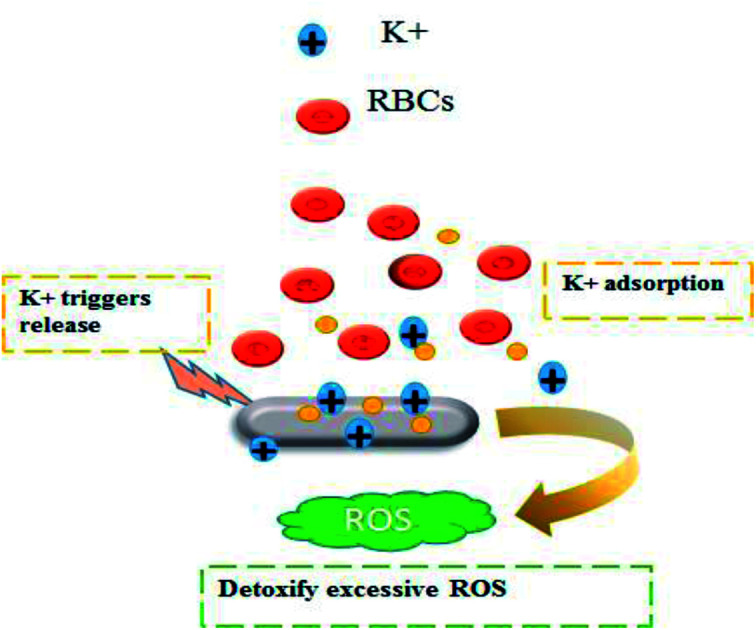

## 1 Introduction

Blood-contacting biomaterials have been widely used for blood-based detection and medical devices for cardiovascular disease.^1,2^ Among these, polymer bags for blood preservation are critical life-saving tools. The commercial blood bags are made of polyvinylchloride (PVC) that contains a high amount of plasticizers.^3^ These plasticizers have been reported to leach out from containers and cause toxicity in the liver and kidneys of rodents and patients.^4^ Therefore, there is an urgent to develop new plasticizer-free polymer containers to substitute these toxic PVC blood bags. Styrene-*b*-(ethylene-*co*-butylene)-*b*-styrene elastomer (SEBS) is the product of a hydrogenated polystyrene-*b*-polybutadiene-*b*-polystyrene (SBS) triblock copolymer, and exhibits an excellent oxidative and hydrolytic resistance.^5^ The advantages of SEBS makes it a potential candidate for the preparation of plasticizer-free bags.^6^ However, long-term contact of red blood cells (RBCs) with SEBS induces serious hemolysis, and can cause intrinsic mechanisms for human disease.^7,8^ So far, the hemolysis of RBCs in SEBS containers has become a bottleneck in this replacement progress.^4^

Cation leakage and membrane oxidation of RBCs are the main causes of hemolysis of RBCs.^9,10^ Cation leakage occurs from the first day of storage, and may be responsible for early changes in the stored RBCs, including in the membrane potential, cell volume and morphology.^11^ Cation leakage causes a redistribution of the monovalent cations, resulting in the loss of internal potassium and the enhancement of potassium in the external media. For example, after 35 days storage the extracellular solution of a leukodepleted unit of packed RBCs contains ∼40–50 mM potassium.^9^ The excess potassium in the blood leads to inactivity of the Na/K-pump and hyperkalaemia.^12^ In addition to cation leakage, membrane oxidation by increased reactive oxygen species (ROS) occurs.^13^ ROS encompass a wide variety of diverse chemical species including superoxides, hypochlorite, hydroxyl anions, and hydrogen peroxide.^14,15^ ROS levels increase gradually for the first week of storage and then rapidly increase to a maximum by the second week of storage. ROS induce damage to the membrane lipids and proteins, reduces the deformability and increases the rigidity of the RBCs. Thus, decreasing cation leakage and membrane oxidation of RBCs is a key strategy to fundamentally improving the quality of preserved RBCs.

Conventional methods based on surface modification have only slight effects on the cation leakage and oxidative injury to RBCs.^16,17^ Considering the suspension of RBCs and the interactions between the RBCs and environmental fluids, maintaining the cellular function using the environmental medium is highly desirable. Controlled release of lecithin and d-α-tocopheryl polyethylene glycol 1000 succinate on the surface of SEBS have been reported to reduce the hemolysis of RBC substantially.^18,19^ However, the release of biomolecules is not responsive to physiological changes in the medium and early release may disturb the metabolism and normal function of the cells. Recently, crown ether-based copolymers have been synthesized for rapid K^+^-triggered drug release.^20–22^ These polymers exhibit K^+^-responsiveness and biocompatibility, which make them suitable for the construction of a smart surface for the cation leakage and membrane oxidation of RBCs. Based on the advantages of electrospinning for encapsulation of biomolecules,^23–25^ we encapsulate the anti-oxidants, 2-*O*-α-d-glucopyranosyl-l-ascorbic acid (AA-2G), in the crown ether nanofibers using an electrospinning technique.

Herein, we electrospun AA-2G-loaded polycaprolactone (PCL)-crown ether nanofibers onto the SEBS surface. Our strategy is based on the sensitive response of the crown ether to leaked potassium causing AA-2G release, the released AA-2G then removes the excess ROS to maintain the Na/K-pump activity and the cell integrity. We demonstrate that AA-2G-loaded PCL-crown ether nanofibers have been well prepared on the surface of SEBS; the nanofibers demonstrate a sensitive response to the excess K^+^ and trigger the rapid release of AA-2G; AA-2G acts as the antioxidant to reduce the excess ROS and maintain the Na/K-pump activity to mitigate cation leakage, resulting in a reduced hemolysis of the preserved RBCs. Our work provides a novel method for the development of plasticizer-free polymers for storage of RBCs, which could be used to fabricate long-term anti-hemolytic biomaterials for use *in vivo*.

## 2 Materials and methods

### 2.1 Materials

A SEBS copolymer with 29 wt% styrene (Kraton G 1652) was provided by Shell Chemicals. PCL, with an average *M*_n_ = 80 000 g mol^−1^ was purchased from Sigma-Aldrich. 4-Nitrobenzo-18-crown-6-ether (NBCE, with an average *M*_n_ = 356 g mol^−1^) was purchased from TCI. Poly(ethylene glycol)diacrylate (PEGDA with an average *M*_n_ = 1000 g mol^−1^) was obtained from Sigma-Aldrich. 1,8-Diazabicyclo-[5.4.0]-7-undecene (DBU, 98%) was purchased from Adamas Reagent Ltd. Benzophenone (BP) was provided by Peking Ruichen Chemical (China). AA-2G, *M*_w_ = 338.27 g mol^−1^ was supplied by Tokyo Chemical Industry (Japan). The chloroform, dimethylformamide (DMF), triethylamine, hydrazine hydrate, acryloyl chloride, dioxane, methylene chloride, ethyl ether, *n*-hexane and toluene used were all reagent grade products. Other reagents were AR-grade and used without further purification. Phosphate-buffered saline (PBS 0.9% NaCl, 0.01 M phosphate buffer, pH 7.4) was freshly prepared.

### 2.2 Synthesis of acylated PCL

Acylated PCL (PCL-A) was synthesized by conjugating the acrylate onto the terminal hydroxyl groups of PCL.^26^ In brief, 4.005 g of PCL was dissolved in 100 mL of toluene solvent under flowing argon gas to displace oxygen. The solution was further degassed using three freeze-pump–thaw cycles, followed by addition of 50 μL of triethylamine. The solution was added dropwise to 10 mL of toluene containing acryloyl chloride (10 μL) for 30 min and was reacted for 24 h in the dark. Finally, the acylated PCL was extracted against *N*-hexane, and dried under vacuum. By comparing the differences between the two groups of peaks, it was confirmed that the acylation reaction was successful. Comparing the ^1^H NMR spectra for PCL and PCL-A, corresponding characteristic peaks were observed at 5.8–6.6 ppm after esterification, and a 3H attributable to the polycaprolactone methyl groups was also found. The acylation of PCL did not affect the hydrophobicity.

### 2.3 Synthesis of benzo-18-crown-6-acrylamide

The synthesis of benzo-18-crown-6-acrylamide (BCAm) was based on 4-nitro-benzo-18-crown-6-ether (NBCe), which was reduced to 4-amino-benzo-18-crown-6-ether (ABCe) using hydrated hydrazine, followed by acylation. Firstly, 2 g of NBCe and 0.2 g of palladium carbon catalyst (Pd/C) were dissolved in 20 mL of fresh steamed dioxane, and were heated at 105 °C in an oil bath to generate reflux. Then, 10 mL of hydrazine hydrate was added dropwise at a constant pressure for 15 min, followed by reaction for 4 h. After cooling to room temperature, the catalyst was removed by filtration and extracted three times with 20 mL deionized water and 20 mL DCM. The product ABCe was obtained by rotary evaporation at 30 °C, and vacuum dried at room temperature for 48 h. Finally, the prepared ABCe was dissolved in DCM, then the acryloyl chloride and triethylamine were added. After reaction at a low temperature for 14 h in the dark, the products were extracted with deionized water and DCM, purified using column chromatography, and rotary evaporated at 20 °C for 48 h. The synthesized polymer and its responsiveness to K^+^ were characterized using a ^1^H NMR spectrometer (Bruker AV 400 MHz) in CDCl_3_. BCAm was dissolved in a deuterated chloroform solution at a concentration of 4 × 10^−2^ M and KNO_3_ solution in deuterated DMSO at a concentration of 0.4 M was added at various ratios of BCAm to K^+^.

### 2.4 Fabrication of K^+^-responsive micro/nanofibers by electrospinning

A mixture of chloroform and DMF (60/40 wt%) was used as the solvent and was incorporated into the previously weighed dry mixture of PCL-A/BCAm/AA-2G at different ratios, with a constant total concentration of polymer/solution of 15 wt%. Then, 2 mL of a solution containing 1 wt% BP was dropped into 4 mL of the mixed solution of chloroform and DMF. Finally, the mixed solutions were transferred to a syringe for electrospinning under UV irradiation. PEGDA was used as the crosslinker (1 wt%) and three compositions were studied: PCL-A, PCL-A/BCAm (3/1 wt%), and PCL-A/BCAm/AA-2G (3/1/1 wt%). The micro/nanofibers were electrospun onto the surface of SEBS at room temperature, a solution feed rate of 0.8–1 mL h^−1^ was used with an applied voltage of 12.5–14.5 kV. The distance between the needles and the collector was approximately 14 cm. For simplicity, the SEBS coated with electrospun fibers was referred to as “electrospun SEBS”.

The morphology of the electrospun SEBS was characterized using field-emission scanning electron microscopy (FESEM, Sirion-100, FEI, U.S.A.). The surface wettability of SEBS and the electrospun SEBS (∼300 μm thickness) was evaluated using the sessile drop method with a pure water droplet (*ca.* 3 μL) using a contact angle goniometer (DSA, KRUSS GMBH, Germany). The surface composition was determined *via* X-ray photoelectron spectroscopy (XPS) by using a VG Scientific ESCA MK II Thermo Avantage V 3.20 analyzer with an Al/K (*hν* = 1486.6 eV) anode mono-X-ray source.

### 2.5 AA-2G release

The electrospun meshes with a size of 1 × 1 cm (100 mg) were incubated in deionized water (11 mL), 10 mM KCl and 15 mM KCl solutions, respectively. Then, at the desired time, 70 μL of solution was collected, and the amount of the released AA-2G was measured using a TECAN instrument (TECAN GENIOS, Austria) operating at 285 nm with a standard calibration curve. The release profile was normalized to the amount of AA-2G initially loaded into the micro/nanofibers.

### 2.6 Preservation of red blood cells

Fresh blood extracted from a healthy rabbit was immediately mixed with 3.8 wt% sodium citrate solution at a dilution ratio of 9 : 1. The experiments were performed in strict accordance with the NIH guidelines for the care and use of laboratory animals (NIH Publication no. 85-23 Rev. 1985) and were approved by the Institutional Animal Care and Use Committee of the Chinese Academy of Sciences (Beijing, China). The whole blood was then centrifuged at 1000 rpm for 15 min to separate the RBCs, white blood cells, and platelet rich plasma. The plasma and buffy coat layers (platelets and white cells) were carefully removed to obtain the concentrated RBCs (100% hematocrit). The virgin and electrospun SEBS films (4 × 4 cm) were then made into 0.4 mL bags, respectively. After sterilization with ethanol for 24 h and drying, 0.2 mL RBCs were transferred into the bags and preserved at 4 °C after sealing.

### 2.7 Determination of K^+^ concentration

The K^+^ concentration in different blood bags was measured after 4 days of storage. The sample was treated with ethanol to precipitate the protein, then reacted with sodium tetraphenylborate (Na-TPB) to generate turbidity and a stable suspension. The turbidity is proportional to the concentration of potassium ions in the sample. Each group of blood bag K^+^ was determined using a commercial assay kit produced by Jian Cheng (Trace potassium ion determination kit, China). Results were expressed as a histogram of the K^+^ concentration mM per liter.

### 2.8 Na/K-ATPase activity

ATPase is present in tissues, cells and organelle membranes, and can decompose ATP to produce ADP and inorganic phosphorous.^27^ Thus, the ATPase activity was determined by the content of Pi. The Na/K-ATPase activity of the stored RBCs was determined with a commercial assay kit produced by Jian Cheng (Ultra-micro sodium and potassium ATPase test kit, China). Results were expressed as a histogram of the Na/K-ATPase activity with a value of the μmol Pi per mL per hour.

### 2.9 Hemolysis and mechanical fragility of the preserved RBCs

A 90 μL sample of the preserved RBCs were collected for hemolysis tests after 4 and 8 days of storage, respectively. The preserved RBCs were diluted with 1 mL normal saline and centrifuged (3000 rpm, 3 min) to obtain the supernatant. Then, the supernatant was transferred to 96-well plates. Positive and negative controls were produced by adding 90 μL fresh RBCs to 1 mL distilled water and normal saline, respectively. After 2 h incubation, the RBCs were removed by centrifugation (3000 rpm, 3 min) and the supernatant was transferred to 96-well plates. The optical density (OD) of the supernatant was measured using a TECAN absorbance reader (TECAN GENIOS, Austria) at 541 nm. The hemolysis ratio (HR) was calculated according to the following formula:1
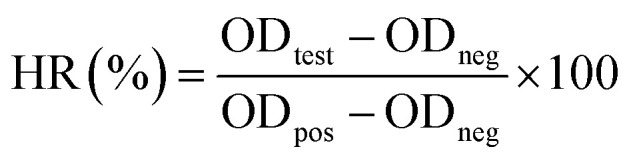
In which OD_test_ is the absorbance value of the test samples, and OD_pos_, and OD_neg_ are the positive (water) and negative (saline) control, respectively.

The mechanical fragility (MF) test was performed according to the method developed by Raval *et al.*^28^ Briefly, 300 μL of preserved RBCs were diluted with 6 mL normal saline and then transferred equally to six tubes (1.8 mL), three of which contained two ellipsoid magnetic stirrers (6 × 10 mm) and three of which did not. The tubes with stirrers were strongly shaken on a thermostatic oscillator for 2 h, and the remaining tubes without stirrers were not shaken and served as a control to ascertain the initial concentration of the free hemoglobin (Hb) in each aliquot. After shaking, all of the tubes were centrifuged twice. For comparison, the MF tests for fresh RBCs were performed under the same conditions. In addition, 50 μL of fresh RBCs were diluted with 1 mL distilled water and incubated for 2 h. The free Hb concentrations in the supernatants were determined using a TECAN absorbance reader (TECAN GENIOS, Austria) at 541 nm. The MF index (MFI) was then calculated according to the following formula:2
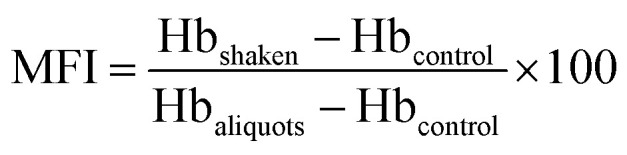
In which Hb_shaken_ is the mean free Hb concentration in the supernatants of the shaken specimens, Hb_control_ is the average free Hb concentration in the supernatants of the control samples, and Hb_aliquot_ is the average Hb concentration in the supernatants of 50 μL fresh RBCs in 1 mL of distilled water after a 2 h treatment.

### 2.10 RBC morphology

The fresh RBCs and preserved RBCs were dropped onto poly-l-lysine-coated glass slides. These RBCs were then incubated at 37 °C for 60 min under static conditions to allow them to adhere firmly onto the glass slides. After the incubation, the samples were carefully rinsed twice with pre-warmed PBS, followed by being immersed in 2.5 vol% glutaraldehyde in PBS (3 mL) for 10 h at 4 °C to fix the adhered RBCs. Finally, the samples were freeze-dried. The morphologies of the adhered RBCs on the sample surfaces were visualized using SEM (SEM, JEOL, JSM-7500F, JP).

### 2.11 Statistical analysis

The degree of hemolysis and oxidation are given as means ± SD for the indicated number of fresh and preserved RBCs. Statistical analysis was performed using Origin Software, with post hoc analysis using Bonferroni's multiple comparison tests when appropriate. Differences were considered statistically significant at *P* ≤ 0.05.

## 3 Results and discussion

### 3.1 K^+^-responsive surface on SEBS

**Fig. 1:**
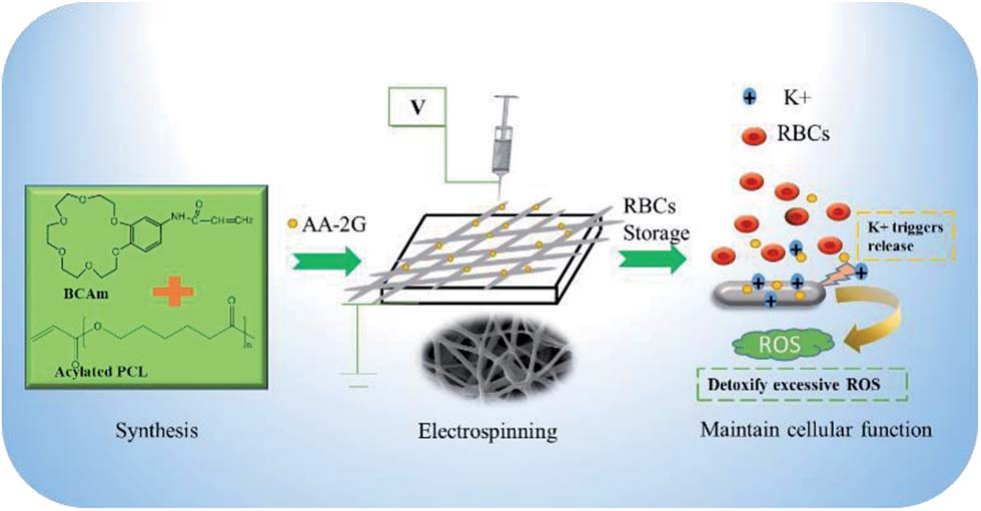
Schematic diagram of the construction of the K^+^-responsive and anti-hemolytic surface on the SEBS substrate by combined functional polymer synthesis and reactive electrospinning.

The RBCs preservation leads to an increase of the extracellular ROS concentration and potassium ion (K^+^) concentration.^9^ Excess ROS and K^+^ deteriorate the activity of Na/K-ATPase, induce membrane oxidation and shorten the life-span of RBCs.^6^ Crown-ether-based copolymers exhibit a high sensitivity to K^+^ (∼5 μM),^20^ the K^+^-responsive surface is constructed for hemolysis reduction in this work. Firstly, PCL-A and BCAm were synthesized. Then, PCL-A and BCAm were mixed with anti-oxidant, AA-2G, and crosslinking agent, PEGDA in a solution of chloroform and dimethylformamide. AA-2G-loaded PCL-BCAm fibers on the SEBS substrate were fabricated by electrospinning. Finally, the electrospun SEBS was made into bags for RBCs storage at 4 °C without adding any blood preservative solution. When the accumulation of K^+^ is over 5 μM, the crown ether responses sensitively and releases AA-2G in a controlled manner. The released AA-2G removes the excess ROS and maintains the Na/K-pump activity to mitigate cation leakage, resulting in the reduced hemolysis of the preserved RBCs (Fig. 1).

### 3.2 Construction and synthesis of PCL-A and BCAm

**Fig. 2:**
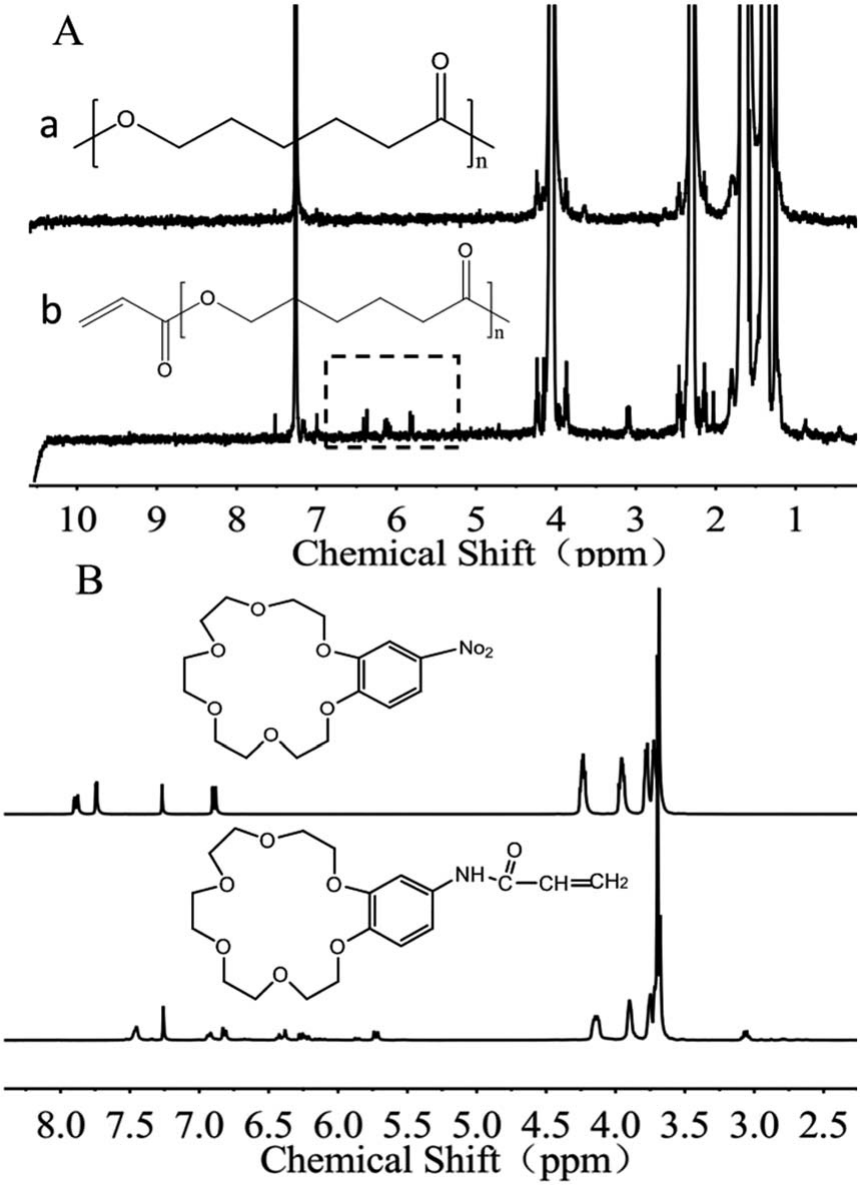
^1^H-NMR spectra of PCL-A (A) and BCAm (B).

PCL acylation was performed by conjugating acrylate onto the terminal hydroxyl groups of PCL (Fig. S1, ESI†). The successful acylation was confirmed using ^1^H-NMR spectra (Fig. 2A). The degree of acylation is determined by comparing the relative peak area of the acryl protons of PCL-A (5.8–6.6 ppm) with that of the three protons of the methyl group in the propylene oxide unit (1.0 ppm) in the ^1^H-NMR spectra.^29^ The degree of resulting acylation was 80–95%. The unsaturated groups on the terminal part of PCL-A provide the active sites for binding the BCAm during electrospinning.

BCAm was synthesized in two steps. Firstly, the nitro (–NO_2_) group connected with the benzene ring of 4-nitrobenzo-18-crown-6-ether (NBCe) was reduced to amidogen (–NH_2_) by hydrazine in the presence of Pd/C catalysts. Then, the reduced NBCe reacted with acryloyl chloride to generate BCAm. The successful synthesis of BCAm is confirmed using ^1^H-NMR spectra. Compared with the NBCe spectrum, the BCAm spectrum exhibits a small chemical shift to a high field, and the apparent acryloyl group peaks (5.8–6.6 ppm) are detected in the BCAm spectrum. In addition, the peaks at 4.16, 3.9, and 3.7 ppm are attributed to the crown ether ring, which responds to K^+^ through complexion.^22^

**Fig. 3:**
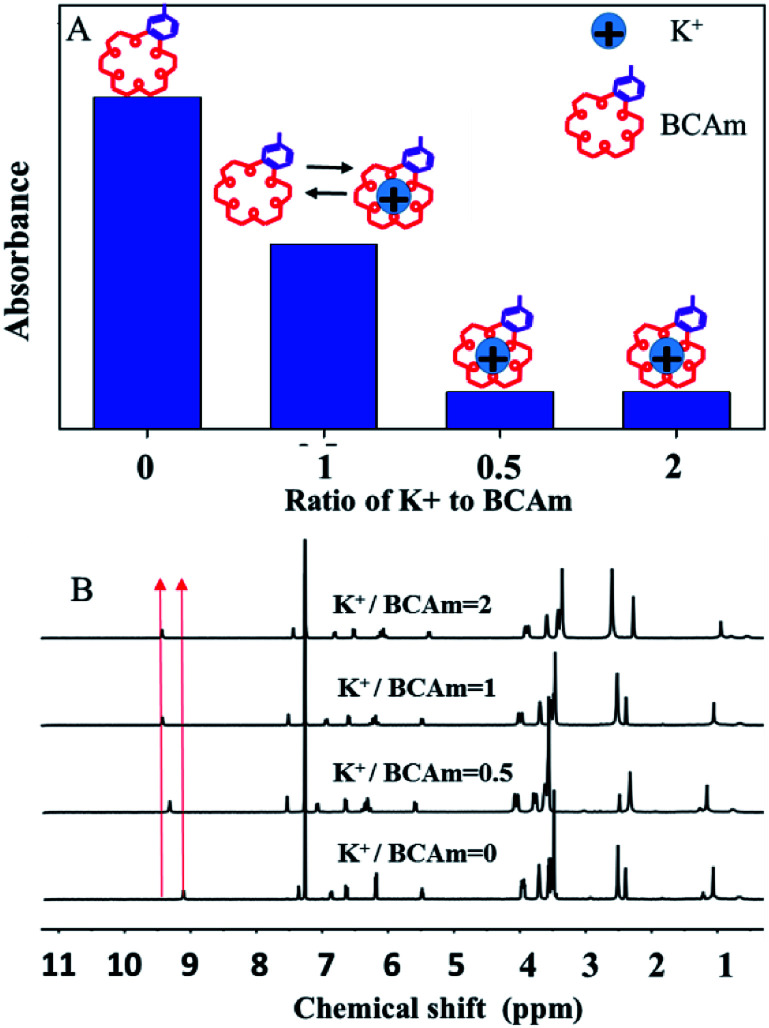
Schematic illustration of K^+^/BCAm complexion (A) and the ^1^H-NMR spectra of K^+^/BCAm complexion (B).

The complexion between BCAm and K^+^ depends on the ratio of BCAm to K^+^ (Fig. 3A). The complexion is quantitatively analyzed using ^1^H-NMR spectra (Fig. 3B). A chemical shift at 9.18 ppm towards the higher field is observed depending on the ratio of BCAm to K^+^. The maximal shift occurs at 9.48 ppm when BCAm/K^+^ = 1, and a similar shift occurs when BCAm/K^+^ = 2. This indicates that the complexion is stable when the molar concentration of BCAm and K^+^ is equal (Fig. 3A). The complexion between BCAm and K^+^ is the molecular basis for the K^+^-responsiveness of the BCAm-based copolymers.^30^

### 3.3 Surface structure and properties of electrospun fibers

PCL-A, BCAm and AA-2G were mixed in a solvent of chloroform and dimethylformamide (60/40 wt%). Then, a solution containing 1 wt% BP and 1 wt% PEGDA was added to the mixture to allow electrospinning under UV irradiation. The UV-induced cross-linking reactions occur during the electrospinning process. The bonding of PCL-A to BCAm is confirmed using the FTIR spectra (Fig. S2, ESI†). The –C=O absorption peak of PCL-A (1729 cm^−1^) and the –N–H stretching vibration peak of BCAm (1664 and 1607 cm^−1^),^31^ are observed in the FTIR spectra of the PCL-A/BCAm fibers. For comparison, PCL-A and PCL-A/BCAm (3/1 wt%) were electrospun using the same conditions.

**Fig. 4:**
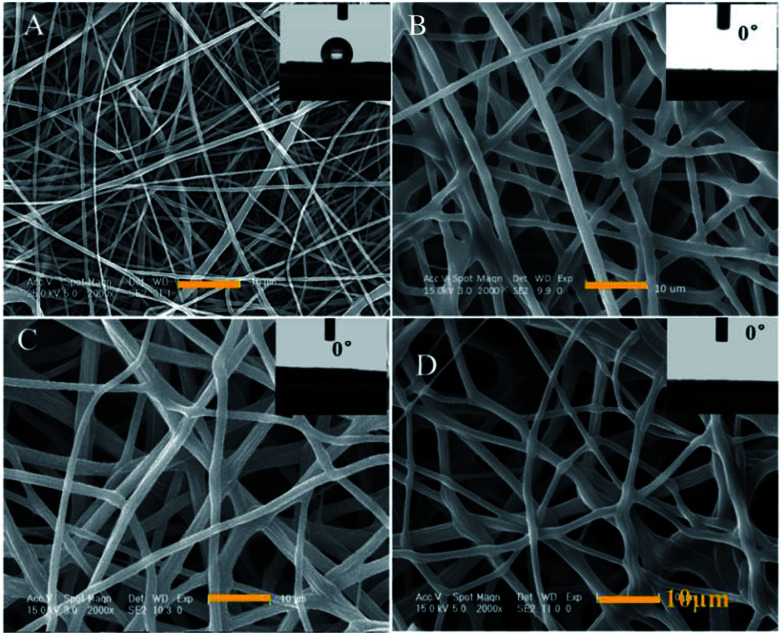
SEM images of electrospun fibers and water contact angles on the surface of the fibers: (A) PCL-A, (B) non-crosslinked PCL-A/BCAm, (C) crosslinked PCL-A/BCAm, and (D) PCL-A/BCAm/AA-2G. Insets are images of water contact angles.

The diameter of the PCL-A microfibers is about 1 μm and exhibits hydrophobicity with a water contact angle (WCA) of 121° (Fig. 4A). In the presence of BCAm, the microfibers become uniform and no beads are observed. The surface is hydrophilic with a WCA of 0° (Fig. 4B). The BCAm molecules are bonded to the PCL chains through PEGDA-associated crosslinking reactions, and the crosslinking does not change the superhydrophilicity of the electrospun meshes (Fig. 4C). The hydrophilicity enables the surface to be hemocompatible.^6^ The introduction of AA-2G has a slight effect on the morphology of the electrospun fibers and the surface remains hydrophilic with a WCA of 0° (Fig. 4D).

### 3.4 K^+^ adsorption and AA-2G release with K^+^ responsiveness

**Fig. 5:**
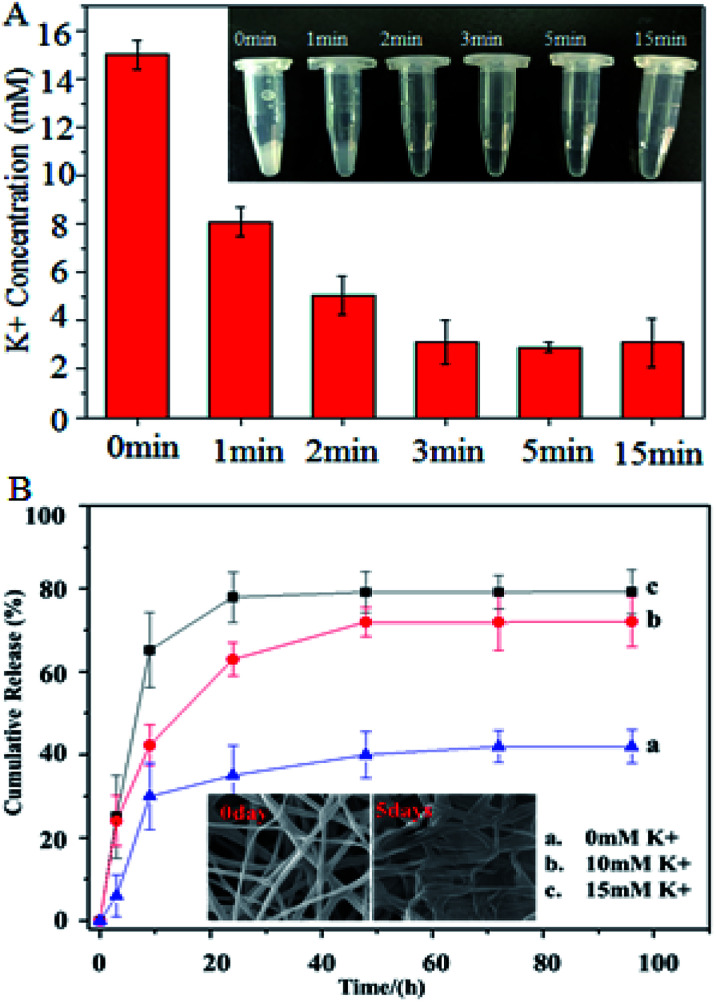
(A) K^+^ adsorption by PCL-A/BCAm/AA-2G nanofibers. (B) Cumulative release of AA-2G from PCL-A/BCAm/AA-2G nanofibers in deionized water (a), 5 mM K^+^ (b), and 15 mM K^+^ (c), respectively. The inset is a photograph of the K^+^ solution after treatment with nanofibers at different amounts of time.

The K^+^ adsorption of the PCL-A/BCAm/AA-2G nanofibers is shown in Fig. 5A, the fiber surface absorbs K^+^ and responds quickly in less than 1 min. The K^+^ solution becomes transparent after treatment with the nanofibers for 15 min (inset of Fig. 5A), indicating that the nanofibers facilitate the reduction of the K^+^ concentration to almost the normal level in the extracellular fluid. The dependence of the release of AA-2G on the K^+^ concentration is shown in Fig. 5B. Only about 42% of the loaded AA-2G is released in 70 hours in deionized water. The release rate of AA-2G in the 10 mM K^+^ (Fig. 5B, line b) and 15 mM K^+^ solution (Fig. 5B, line c) is much greater than that in the deionized water, illustrating that the AA-2G release rate increases as the K^+^ concentration increases. The crown ethers on the fiber surface capture the K^+^ and render the surface hydrophilic to favor the access of water molecules, resulting in accelerated AA-2G release.^6,22^ The electrospun fibers collapse after the release of AA-2G (insets of Fig. 5B).

### 3.5 Hemolysis reduction

**Fig. 6:**
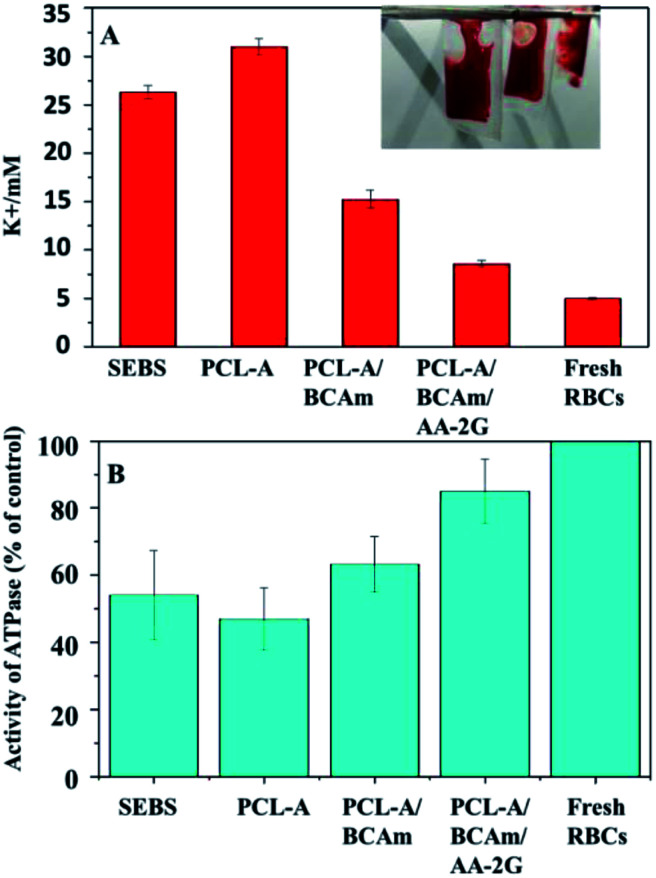
(A) K^+^ concentration in the preserved RBCs, and (B) Na/K-ATPase activity of the preserved RBCs.

To test the efficiency of the anti-hemolytic surface, RBCs were packaged in the bags made of SEBS film and electrospun SEBS and preserved at 4 °C for several days (inset of Fig. 6A). The accumulation of K^+^ in the different bags was measured (Fig. 6A). The concentration of K^+^ in the SEBS bags and the SEBS/PCL-A bags increases significantly after 4 days of storage. In contrast, the concentrations of K^+^ in the PCL-A/BCAm and PCL-A/BCAm/AA-2G bag decrease substantially. The concentrations of K^+^ is close to normal levels (∼5 mM) in the PCL-A/BCAm/AA-2G bag, indicating that the K^+^-responsive surface mitigates the cation leakage and provides a normal environment for cellular function.^9^

Na/K-ATPase hydrolyzes an ATP molecule to export 3Na^+^ ions in exchange for the importing of 2K^+^ ions. It constantly corrects the cation leakage and maintains a gradient of ion concentration in the RBCs.^32^ The Na/K-ATPase activity was characterized to evaluate the cellular integrity (Fig. 6B). The Na/K-ATPase activity of the RBCs in the bags made of PCL-A/BCAm and PCL-A/BCAm/AA-2G are much higher than that in the blood bags made from SEBS and PCL-A. As the high concentration of K^+^ and oxidation damages the Na/K-ATPase,^9^ the high activity of Na/K-ATPase demonstrates the reduced K^+^ concentration and oxidation, resulting in reduced hemolysis.

**Fig. 7:**
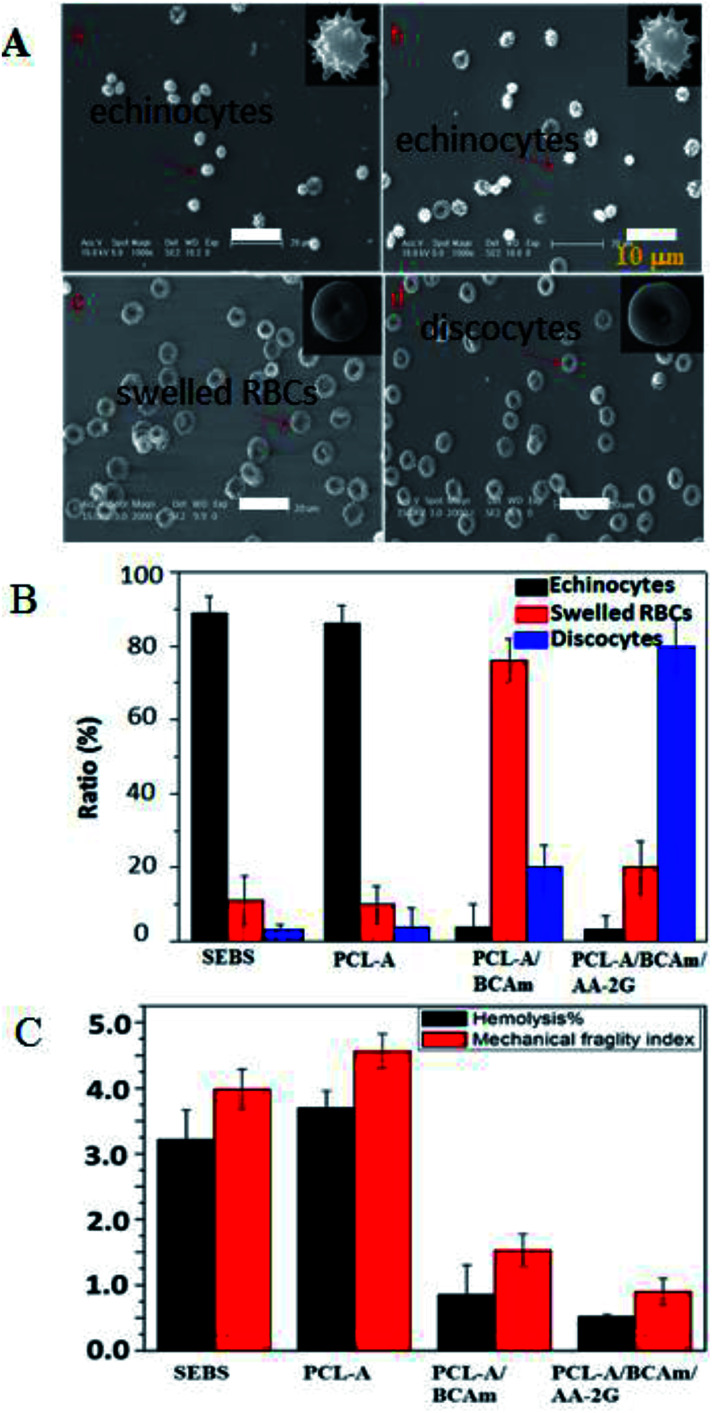
(A) Morphology of RBCs in bags made from SEBS (a), PCL-A (b), PCL-A/BCAm (c), and PCL-A/BCAm/AA-2G (d). (B) Statistical analysis the ratio of the morphology of RBCs. (C) The hemolysis and mechanical fragility of the persevered RBCs.

The morphology of the preserved RBCs was observed and shown in Fig. 7A. The fresh RBCs have the appearance of regular biconcave discs without any damage. Most of the preserved RBCs in the SEBS bags (Fig. 7A-a) and the PCL-A bags (Fig. 7A-b) are irregular echinocytes that have many protrusions, and exhibit irreversible deformation. In contrast, most of the preserved RBCs in the electrospun PCL-A/BCAm bag are regular biconcave discs (Fig. 7A-c), indicating that the reduction of the K^+^ concentration favors normal cellular function.^33^ The shape of the regular biconcave discs is dominant in the PCL-A/BCAm/AA-2G bags (Fig. 7A-d), confirming that the cellular normal function can be maintained. The statistical analysis of the RBC morphology is exhibited in Fig. 7B, echinocytes are dominant in the SEBS and PCL-A bags (>85%), while only a few echinocytes are observed in the PCL-A/BCAm bags (<5%), indicating that cation leakage is one of the key factors for hemolysis. In contrast, discocytes are dominant in the PCLA/BCAm/AA-2G bags. It is well known that changes in the shape of RBCs are determined by the membrane deformability,^34^ and regular biconcave-RBCs confirm the high deformability of the RBCs. Therefore, the advantages of the K^+^ responsive surface are further demonstrated by the hemolysis and the MFI of the RBCs in the different bags (Fig. 7C). After 4 days of storage, the hemolysis ratio and the MFI of the preserved RBCs decreases, going in the order SEBS to PCL-A, PCL-A/BCAm and the PCLA/BCAm/AA-2G bags. The above results demonstrate that the constructed surfaces reduce the cation leakage and oxidative injury to RBCs. As the response of the fibers can be tuned to other physiological stimuli,^35^ this method is facile and versatile, and provides a novel method for the preparation of anti-oxidative implant biomaterials for use *in vivo*.

## 4 Conclusions

In summary, we fabricated AA-2G-loaded PCL-crown ether micro/nanofibers on a SEBS surface to prevent cation leakage and membrane oxidation of the preserved RBCs. Our strategy was based on the sensitivity of the crown ether sensitivity to the leaked potassium, enabling AA-2G release and this released AA-2G could then remove the excess ROS to maintained the Na/K-ATPase activity and the cell integrity. We demonstrated that AA-2G-loaded PCL-crown ether nanofibers were well prepared on the surface of SEBS; the nanofibers responded sensitively to the excess K^+^ and triggered the raid release of AA-2G. AA-2G acted as an antioxidant to reduce the excess ROS and maintain the Na/K-pump activity to mitigate cation leakage, resulting in the reduced hemolysis of the preserved RBCs. Our work provides a novel method for the development of plasticizer-free polymers for the storage of RBCs, which could potentially be used to fabricate long-term anti-hemolytic biomaterials for use *in vivo*.

## Conflicts of interest

There are no conflicts to declare.

## Supplementary Material

RA-009-C8RA08215D-s001

## References

[cit1] Werner C., Maitz M. F., Sperling C. (2007). Current strategies towards hemocompatible coatings. J. Mater. Chem..

[cit2] Biran R., Pond D. (2017). Heparin coatings for improving blood compatibility of medical devices. Adv. Drug Delivery Rev..

[cit3] Simmchen J., Ventura R., Segura J. (2012). Progress in the removal of di-[2-ethylhexyl]-phthalate as plasticizer in blood bags. Transfus. Med. Rev..

[cit4] Ishihara K., Ishihara K., Nishiuchi D., Watanabe J., Iwasaki Y. (2004). Polyethylene/phospholipid polymer alloy as an alternative to poly(vinylchloride)-based materials. Biomaterials.

[cit5] Parameswaranpillai J., Joseph G., Shinu K. P., Jose S., Salim N. V., Hameed N. (2015). Development of hybrid composites for automotive applications: effect of addition of SEBS on the morphology, mechanical, viscoelastic, crystallization and thermal degradation properties of PP/PS–*x*GnP composites. RSC Adv..

[cit6] Wang H. Z., Xu X. D., Chen R. H., Zhao J. R., Cui L. L., Sheng G. K., Shi Q., Wong S., Yin J. H. (2017). Bioinspired antioxidant defense system constructed by antioxidants-eluting electrospun F127-based fibers. ACS Appl. Mater. Interfaces.

[cit7] Medzhitov D. S. S. R., Soares M. P. (2012). Disease tolerance as a defense strategy. Science.

[cit8] Schaer D. J., Buehler P. W., Alayash A. I., Belcher J. D., Vercellotti G. M. (2013). Hemolysis and free hemoglobin revisited: exploring hemoglobin and hemin scavengers as a novel class of therapeutic proteins. Blood.

[cit9] Flatt J. F., Bawazir W. M., Bruce L. J. (2014). The involvement of cation leaks in the storage lesion of red blood cells. Front. Physiol..

[cit10] Liu W. F., Ma M., Bratlie K. M., Dang T. T., Langer R., Anderson D. G. (2011). Real-time *in vivo* detection of biomaterial-induced reactive oxygen species. Biomaterials.

[cit11] Berezina T. L., Zaets S. B., Morgan C., Spillert C. R., Kamiyama M., Spolarics Z., Deitch E. A., Machiedo G. W. (2002). Influence of storage on red blood cell rheological properties. J. Surg. Res..

[cit12] Udensi U. K., Tchounwou P. B. (2017). Potassium homeostasis, oxidative stress, and human disease. Int. J. Clin Exp. Physiol..

[cit13] Yoshitomi T., Yamaguchi Y., Kikuchi A., Nagasaki Y. (2012). Creation of a blood-compatible surface: a novel strategy for suppressing blood activation and coagulation using a nitroxide radical-containing polymer with reactive oxygen species scavenging activity. Acta Biomater..

[cit14] Huie R. E. and Neta P., Chemistry of reactive oxygen species, Springer, 2002, vol. 2, p. 17

[cit15] Melov S., Ravenscroft J., Malik S., Gill M. S., Walker D. W., Clayton P. E. (2000). Extension of life-span with superoxide dismutase/catalase mimetics. Science.

[cit16] Yang W. J., Neoh K. G., Kang E. T., Teo S. L. M., Rittschof D. (2014). Polymer brush coatings for combating marine biofouling. Prog. Polym. Sci..

[cit17] Li C., Jin J., Liu J., Xu X., Yin J. (2014). Improving hemocompatibility of membranes for extracorporeal membrane oxygenators by grafting nonthrombogenic polymer brushes. RSC Adv..

[cit18] Shi Q., Fan Q., Ye W., Hou J., Wong S. C., Xu X., Yin J. (2014). Controlled lecithin release from a hierarchical architecture on blood-contacting surface
to reduce hemolysis of stored red blood cells. ACS Appl. Mater. Interfaces.

[cit19] Shi Q., Fan Q., Hou J., Wong S. C., Xu X., Yin J. (2015). Construction of d-α-tocopheryl polyethylene glycol succinate/PEO core–shell nanofibers on a blood-contacting surface to reduce the hemolysis of preserved erythrocytes. J. Mater. Chem. B..

[cit20] You X. R., Ju X. J., He F., Wang Y., Liu Z., Wang W., Xie R., Chu L. Y. (2017). Polymersomes with rapid K(+)-triggered drug-release behaviors. ACS Appl. Mater. Interfaces.

[cit21] Taichi Ito T. H., Yamaguchi T., Shinbo T., Nakao S., Kimura S. (2002). Development of a molecular recognition ion gating membrane and estimation of its pore size control. J. Am. Chem. Soc..

[cit22] Yagi K., Ruiz J. A., Sanchez M. C. (1980). Cation binding properties of polymethacrylamide derivatives of crown ethers. Makromol. Chem..

[cit23] Ding B., Wang M., Wang X., Yu J., Sun G. (2010). Electrospun nanomaterials for ultrasensitive sensors. Mater. Today.

[cit24] Shi Q., Hou J., Zhao C., Xin Z., Jin J., Li C., Wong S.-C., Yin J. (2016). A smart core–sheath nanofiber that captures and releases red blood cells from the blood. Nanoscale.

[cit25] Amariei G., Kokol V., Boltes K., Letón P., Rosal R. (2018). Incorporation of antimicrobial peptides on electrospun nanofibres for biomedical applications. RSC Adv..

[cit26] Kweon H. Y., Yoo M. K., Park I. K., Kim T. H., Lee H. C., Lee H. S. (2003). A novel degradable polycaprolactone networks for tissue engineering. Biomaterials.

[cit27] Wang F., Bronich T. K., Kabanov A. V., Rauh R. D. (2005). Synthesis and evaluation of a star amphiphilic block copolymer from poly(ε-caprolactone) and poly(ethylene glycol) as a potential drug delivery carrier. Bioconjugate Chem..

[cit28] Raval J. S., Fontes J., Banerjee U., Yazer M. H., Mank E., Palmer A. F. (2013). Ascorbic acid improves membrane fragility and decreases haemolysis during red blood cell storage. Transfus. Med..

[cit29] Shachaf Y., Wadmany M. G., Seliktar D. (2010). The Biocompatibility of Pluronic F127 fibrinogen-based hydrogels. Biomaterials.

[cit30] Zhang B., Ju X. J., Xie R., Liu Z., Pi S. W., Chu L. Y. (2012). Comprehensive effects of metal ions on responsive characteristics of P(NIPAM-*co*-B18C6Am). J. Phys. Chem. B.

[cit31] Wei X., Shao B., He Z., Ye T., Luo M., Sang Y., Liang X., Wang W., Luo S., Yang S., Zhang S., Gong C., Gou M., Deng H., Zhao Y., Yang H., Deng S., Zhao C., Yang L., Qian Z., Li J., Sun X., Han J., Jiang C., Wu M., Zhang Z. (2015). Cationic nanocarriers induce cell necrosis through impairment of Na(+)/K(+)-ATPase and cause subsequent inflammatory response. Cell Res..

[cit32] Castillo J. P., Rui H., Basilio D., Das A., Roux B., Latorre R., Bezanilla F., Holmgren M. (2015). Mechanism of potassium ion uptake by the Na(+)/K(+)-ATPase. Nat. Commun..

[cit33] Hughes M. N. (1999). Relationships between nitric oxide, nitroxyl ion, nitrosonium cation and peroxynitrite. Biochim. Biophys. Acta, Rev. Bioenerg..

[cit34] Chien S. (1987). Annu. Red cell deformability and its relevance to blood flow. Rev. Physio..

[cit35] Escalona G. R., Sanchis J., Vicent M. J. (2018). pH-Responsive polyacetal-protein conjugates designed for polymer masked–unmasked protein therapy (PUMPT). Macromol. Biosci..

