# Supplementary material for: Construction of K+ responsive surface on SEBS to reduce the hemolysis of preserved erythrocytes
Source: RSC Adv. 2019 Feb 11;9(10):5251–8. doi: 10.1039/c8ra08215d (PMC9060672; doi:10.1039/c8ra08215d)
Supplement: RA-009-C8RA08215D-s001 [file RA-009-C8RA08215D-s001.pdf]

Electronic Supplementary Material (ESI) for RSC Advances.
This journal is © The Royal Society of Chemistry 2019

Supporting Information

Construction of K+ responsive surface on SEBS to reduce the hemolysis of preserved erythrocyte

Xingkun Luan,[†,‡] Haozheng Wang,[†] Zehong Xiang,[‡] Jiruo Zhao,[†] Ying Feng,[†,*] Qiang Shi,[‡,*] Shing-Chung Wong,[⊥] Jinghua Yin[†]

[†] Shandong Provincial Key Laboratory of Olefin Catalysis and Polymerization/Key Laboratory of Rubber-Plastics (QUST), Ministry of Education/Shandong, Qingdao 266042, P. R. China

[‡] State Key Laboratory of Polymer Physics and Chemistry, Changchun Institute of Applied Chemistry, Chinese Academy of Sciences, Changchun 130022, P. R. China

[⊥] Department of Mechanical Engineering, University of Akron, Akron, Ohio 44325-3903, USA

Content:

1. Synthesis pathway for PCL-A
2. FTIR spectra of PCL-A, BCAm and electrospun PCL-A/BCAm fiber

1. Synthesis pathway for PCL-A

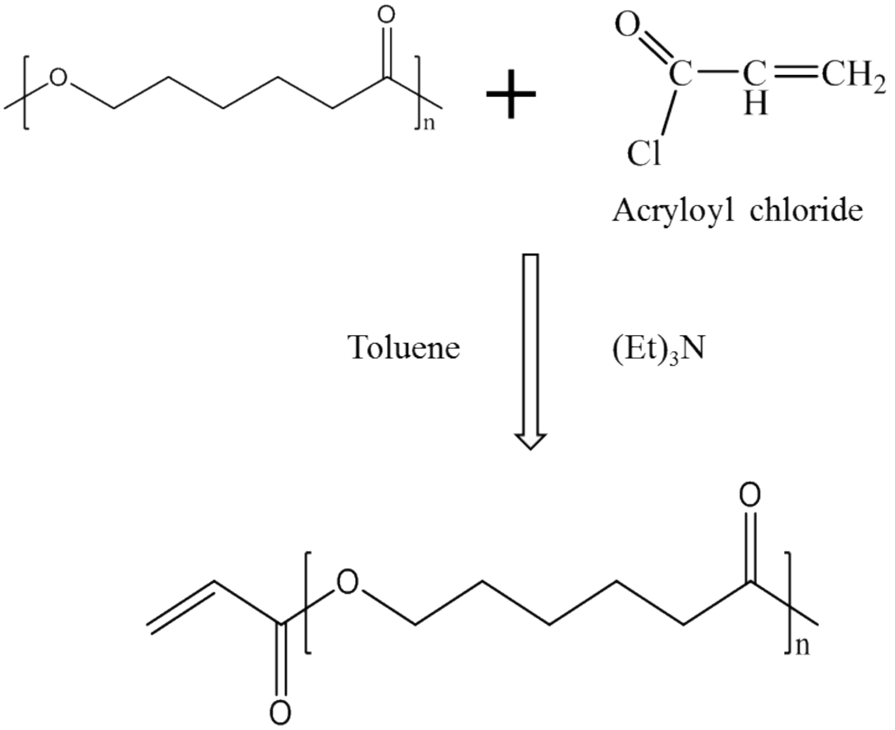

Figure S1. Synthesis pathway for PCL-A

2. FTIR spectra of PCL-A, BCAm and electrospun PCL-A/BCAm fiber

Bruker FTIR spectrometer Vertex 70 equipped with an attenuated total reflection (ATR) unit (ATR crystal 45°) was used to characterize PCL-A, BCAm and electrospun PCL-A/BCAm fiber at a resolution of 4 cm^{-1} for 32 scans. As shown in Figure S2, the peak at 1729 cm^{-1} is attributed to the -C=O absorption of the acrylate group on PCL-A, BCAm shows the peaks at 1664 cm^{-1} and 1607 cm^{-1}, which are assigned to the -C=O absorption and the -N-H stretching vibration peaks, respectively. These peaks can be observed in FTIR spectra of PCL-A/BCAm fibers.

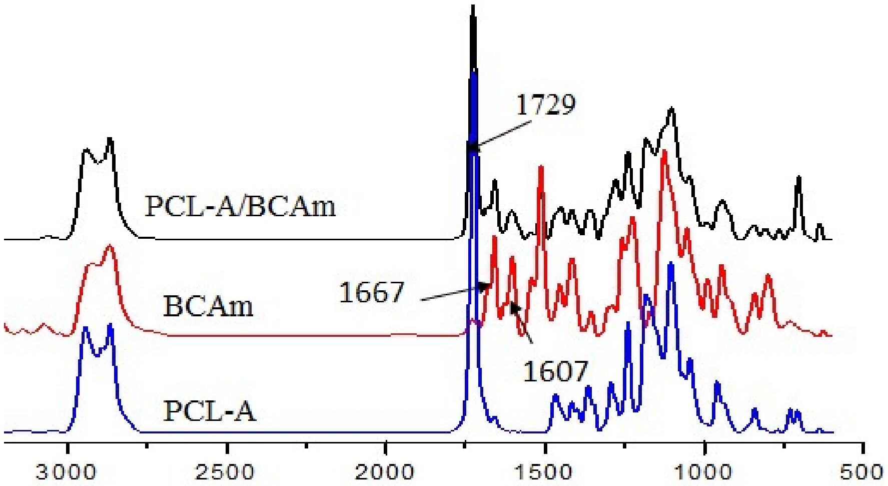

Figure S2. FTIR of PCL-A, BCAm and electrospun PCL-A/BCAm
